# Migraine and Despair: Factors Associated with Depression and Suicidal Ideation among Canadian Migraineurs in a Population-Based Study

**DOI:** 10.1155/2013/401487

**Published:** 2013-10-13

**Authors:** Esme Fuller-Thomson, Meghan Schrumm, Sarah Brennenstuhl

**Affiliations:** ^1^Factor-Inwentash Faculty of Social Work, University of Toronto, Toronto, ON, Canada M5S 1A1; ^2^Dalla Lana School of Public Health, University of Toronto, Toronto, ON, Canada M5T 3M7

## Abstract

This study sought to (1) investigate the association between migraine and both depression and suicidal ideation and (2) to identify the factors independently associated with each of these mental health problems among Canadian men and women with migraine. Data were analyzed from the 2005 Canadian Community Health Survey (CCHS). Presence of migraine was assessed by self-report of a health professional diagnosis. Current depression was measured using the CIDI-SF, and suicidal ideation was based on a question about serious consideration of suicide at any point during the respondent's lifetime. Migraineurs were found to have elevated odds of depression (men: OR = 2.02; 95% CI = 1.70, 2.41; women: OR = 1.89; 95% CI = 1.71, 2.10) and suicidal ideation (men: OR = 1.70; 95% CI = 1.55, 1.96; women: OR = 1.72; 95% CI = 1.59, 1.86) even when adjusting for sociodemographic variables and disability status. The odds of depression and suicidal ideation were higher among both genders of migraineurs who were younger, unmarried and had more activity limitations; associations with poverty and race depended on gender and whether the focus was on depression or suicidal ideation. While screening for depression is already recommended for those with migraine, this research helps identify which migraineurs may require more immediate attention, including those who are younger, unmarried, and experiencing limitations in their activities.

## 1. Introduction 

The recently launched “36 million migraine campaign” underlines the scope of the influence of migraine on the American population, wherein it is estimated that about one in every four households contain someone who experiences migraine [1–3]. Research from other countries confirms the high prevalence of migraine and the large burden it places on the lives of its sufferers [4, 5]. Migraines comprise a group of neurological symptoms that typically include severe, reoccurring, and throbbing pain on at least one side of the head. Individuals who suffer from migraine often experience disruptions in their work, family, social activities, and overall quality of life [6]. Costs related to migraine due to absenteeism and diminished productivity are estimated to be about 20 billion dollars per year in the US [7]. 

A number of population-based studies from North America and Europe have shown that individuals suffering from migraine have between about 1.3 and 5.8 times higher odds of depression than those without this condition [8–15]. The relationship between migraine and depression, however, is likely to be bidirectional [8, 15]. Breslau and colleagues [8, 15] have shown that those reporting depression at baseline have a higher risk of first-onset migraine during the two-year follow-up period and that those with migraine at baseline have an increased risk of developing first onset major depression during followup. These findings suggest that the association between migraine and depression is best explained by shared causes, such as genetic factors or neurochemical abnormalities, rather than, for example, solely a psychological response to the experience of recurring migraine [8, 15–17]. Migraineurs also have higher odds of suicidal behaviours, including attempts and suicidal ideation, than their counterparts without migraine [18–20]. The common etiology hypothesis has also been proposed to explain the relationship between migraine and suicidal behaviours [18], although recent research suggests that the link may be accounted for by pain severity [20]. 

While there is strong evidence of a relationship between migraine and both depression and suicidal behaviours, less is known about what factors are associated with each of these comorbidities and whether they differ for depression and suicidal behaviours. One study found that individuals with migraine who are widowed, separated, or divorced have a significantly higher prevalence of major depressive disorder than those who are single or married/common law [9]. This study also found that a higher percentage of migraineurs with low incomes had depression than their more economically advantaged peers and that those with both migraine and depression had higher rates of disability and restriction of activities [9]. It remains unclear whether these factors are independently associated with depression among those with migraine and whether these factors also relate to suicidal behaviours. The current study sought to achieve two main goals: investigate the association between migraine and each of current depression and lifetime suicidal ideation using a population-based sample and to identify the gender-specific factors that are independently associated with depression and suicidal ideation among those who report that they have been diagnosed with migraine by a health professional. 

## 2. Methods

### 2.1. Data Sources

This study draws on the public use data files of the 3.1 Canadian Community Health Survey (CCHS), conducted in 2005. The CCHS is a nationally representative survey that is generalizable to approximately 98% of all Canadians aged 12 years or older and provides national estimates of “health determinants, health status and health system utilization” [21]. The CCHS used a multistaged stratified cluster design in which the household was the final sampling unit. In selected households, a knowledgeable person was asked to provide basic demographic information for all of its residents. One person per household was then selected for an in-depth interview using varying probabilities that take into account age and the household composition. In 2005, the household-level response rate was 84.9%, which equals to 143,076 of the 168,464 households selected to participate. Fully 92.9% of individuals (*n* = 132, 947) in the participating households responded to the survey. At the national level, these figures yield a combined response rate of 78.9%. This unusually high response rate for a population-based survey is partially due to the fact that the CCHS was conducted by Statistics Canada, a highly respected institution that is the Canadian equivalent of the U.S. Census Bureau.

Unfortunately, questions on depression and suicidal ideation were not asked in all health regions in Canada. Consequently, the current study is based on data from the six provinces that included optional modules on depression and/or suicidal ideation (i.e., Prince Edward Island, Nova Scotia, Quebec, Saskatchewan, Ontario, and Alberta). In these provinces, combined response rates ranged from 76.3% (Quebec) to 84.1% (Saskatchewan).

### 2.2. Sample

Two distinct samples from the CCHS were used for the analysis. The first sample was based on all respondents from the four provinces that screened for depression using a standardized scale (i.e., Prince Edward Island, Nova Scotia, Quebec, and Saskatchewan). Out of the 71,234 respondents from these provinces, 67,674 had valid data on both migraine and depression, representing 95% of the sample in these provinces (31,017 men and 36,657 women). An additional 473 men (1.5%) and 481 women (1.3%) were excluded due to missing data on one or more of the independent variables in the multivariate analysis. A total of 1,801 men and 4,878 women had migraine, and 1,244 men and 2,515 women were depressed. In the analyses restricted to those with migraine, 36 men (2.0%) and 66 women (1.4%) were excluded due to missing data on one or more of the independent variables. 

The second sample was based on respondents from the four provinces where questions on suicidal ideation were asked (Quebec, Ontario, Saskatchewan, and Alberta). Out of the 90,496 respondents from these provinces, 82,619 had complete data on the migraine and suicide questions, representing 91.3% of the sample (37,417 males and 45,202 females). An additional 549 men (1.5%) and 602 women (1.3%) were excluded due to missing data on one or more of the independent variables in the multivariate analyses. In this sample, a total of 2,292 men and 6,439 women had migraine, and 3,526 men and 5,168 women had seriously considered suicide. In the analyses restricted to those with migraine, 37 men (1.6%) and 99 women (1.5%) were excluded due to missing data on one or more of the independent variables. 

### 2.3. Measures

*Current depression* was ascertained using the Composite International Diagnostic Interview-Short Form (CIDI-SF) [22]. Based on the fully-structured CIDI interview, the CIDI-SF is a shortlist of items that measure a major depressive episode using the definitions and the criteria of the Diagnostic and Statistical Manual III and the diagnostic criteria of the International Classification of Diseases. Respondents were classified as depressed if they had a probability of 90% or greater for a major depressive episode over a period of at least two weeks in the last year. The CIDI has excellent interrater reliability and good test-retest reliability and validity. In comparison to CIDI, the total classification accuracy for a major depressive episode of the CIDI-SF is 93.2%. The sensitivity and specificity of the CIDI-SF are 89.6% and 93.9%, respectively.

*Lifetime suicidal ideation* was measured using the question: have you ever seriously considered committing suicide or taking your own life? Note that this question was only asked of respondents aged 15 and older.

*Migraine:* respondents were asked to indicate the long-term health conditions that they had which were expected to last, or had already lasted, six months or more. After reminding the participant to report only conditions that were diagnosed by a health professional, they were asked: “do you have migraine headaches?”

*Sociodemographic characteristics* included age (<30, 30–49, 50–64, ≥65), educational achievement (≤high school graduate versus postsecondary graduate), race, annual household income (<$30,000 versus ≥$30,000; a missing category was also included), and marital status (married/common law versus divorced/separated/never married). All analyses were gender specific due to the knowledge that migraine and depression are both more common among women than men [5, 23]. 

*Disability* was assessed using two measures. The first was limitations in activities caused by a long-term health condition or problem using the question: “Do you have any difficulty hearing, seeing, communication, walking, climbing stairs, bending, leaning, or doing any similar activities (sometimes, often, or never)?” The second was the degree to which the respondent is unable to be independent in their activities of daily living (ADL) based on the question: “Because of any physical condition, mental condition, or health problem, do you need the help of another person with personal care such as washing, dressing, eating, or taking medication?”

### 2.4. Statistical Analyses

The statistical analysis was undertaken in three stages for each of depression and suicidal ideation. First, the percentages of those with migraine and depression/suicidal ideation were calculated based on the full sample. Second, an adjusted odds ratio of depression/suicidal ideation for those with migraine versus those without was estimated. Third, focusing on the subsample of those with migraine, crude and adjusted odds ratios of depression/suicidal ideation were computed for each of the sociodemographic and disability variables. 

In line with the recommendations of Statistics Canada, all percents, means, standard deviations, odds ratios, and prevalence ratios were determined by using standardized CCHS sampling weights to account for the probability of selection [24]. All sample sizes are documented in their unweighted form. SPSS version 20 was utilized to perform the aforementioned analyses. 

## 3. Results

### 3.1. Depression

The first set of results pertains to the sample of those with valid data on depression and migraine. In this sample, about 6.1% of men had migraine compared to 14.1% of women. Also, a greater share of women were depressed 6.6% than men 3.7%. Among both men and women, the prevalence of depression among those with migraine was significantly higher than those without migraine (men: 8.4% versus 3.4%; women 12.4% versus 5.7%; each *p* < .001). 

Table 1 presents the results of the gender-specific logistic regression predicting depression among those with migraine versus those without. For men, the age- and race-adjusted odds of depression among those with migraine were 2.49 times higher than those without migraine (95% CI = 2.10, 2.95). The relationship was attenuated but remained strong and significant when further adjustments were made for marital status, income, education (OR = 2.46; 95% CI = 2.07, 2.91), activity limitations, and ADLs (OR = 2.02; 95% CI = 1.70, 2.41). For women, the age- and race-adjusted odds of depression among those with migraine were 2.17 times higher than those without migraine (95% CI = 1.97, 2.40) and were reduced to 1.89 (95% CI = 1.71, 2.10) in the fully adjusted model. For both men and women, those with depression were younger, unmarried, and poorer and had more limitations in activities and ADLs than the nondepressed. 

The results of the gender-specific logistic regression predicting depression among those with migraine are presented in Table 2. When the sample was restricted to women with migraine, those with depression were younger, unmarried, and poorer and had more activity limitations and limitations in ADLs than the nondepressed. The same factors were associated with depression in the logistic regression of men with migraine.

### 3.2. Suicidal Ideation

The second set of analyses pertains to the sample with valid data on suicidal ideation and migraine. The prevalence of migraine (men: 6.5%; women: 15.3%) was a little higher in this sample. Slightly fewer men than women experienced suicidal ideation (men: 8.4%; women: 10.4%). Among both men and women, suicidal ideation was more common for those with migraine (men: 15.6% versus 7.9%; women: 17.6% versus 9.1%; each *p* < .001). 

Table 3 presents the results of the gender-specific logistic regression predicting suicidal ideation among those with migraine versus those without. For men, the age- and race-adjusted odds of suicidal ideation among those with migraine were twice those without migraine (OR = 2.01; 95% CI = 1.79, 2.25). Further adjustment for marital status, income, and education had no impact on the odds ratio of suicidal ideation (OR = 2.01; 95% CI = 1.79, 2.25). When additional adjustments were made for ADL limitations and activity limitations, the odds ratio declined but remained significant (OR = 1.70; 95% CI = 1.55, 1.96). For women, the age- and race-adjusted odds of suicidal ideation among those with migraine were 1.95 times higher than those without migraine (95% CI = 1.81, 2.10). Further adjustment for marital status, income and education (OR = 1.93; 95% CI = 1.79, 2.08), as well as ADL limitations and activity limitations, (OR = 1.72; 95% CI = 1.59, 1.86) had relatively little influence on the odds ratio.

The results of the gender-specific logistic regression analyses predicting suicidal ideation among those with migraine are presented in Table 4. When the sample was restricted to women with migraine, the odds of suicidal ideation were higher for those who were younger, unmarried, and poorer and had more limitations in activities than those who had never seriously considered suicide. For men with migraine, the odds of suicidal ideation were also higher among younger, unmarried respondents and those with limitations in their activities. However, income was not associated with suicidal ideation among men with migraine, while white racial identity was. Education and ADL limitations were not significantly associated with suicidal ideation in either gender. 

## 4. Discussion

Consistent with previous population-based research from Canada and abroad, this study shows that migraine is associated with current depression and lifetime suicidal ideation among members of the general community, even when accounting for sociodemographic factors and disability status [8–14, 18, 20]. This study also adds to the literature by identifying the gender-specific factors that are independently associated with depression and with suicidal ideation among Canadians with migraine. Among both women and men with migraine, younger age, being unmarried, being poor, and having greater disability were each independently associated with higher odds of depression. One prior study also showed that a higher percentage of those with depression and migraine were poor and unmarried and had greater disability than those with migraine and no depression [9]; however, this research did not investigate whether these factors were independent of one another or use gender-specific analysis. 

No studies that we are aware of have looked at the factors associated with lifetime suicidal ideation among those with migraine. Moreover, we found some evidence of gender variation in these factors. For female migraineurs, most of the same factors that were related to depression were also linked to suicidal ideation (i.e., younger age, being unmarried, being poor, and having greater limitations in activities). For male migraineurs, three of these factors (i.e., younger age, being unmarried, and having greater limitations in activities) were associated with suicidal ideation; however, the other, being poor, was not. Further, white racial identity was associated with higher odds of suicidal ideation only among males with migraine. This is somewhat consistent with the literature, which finds that white males have a higher risk of suicide than other racial groups, especially black males [25]. 

The vulnerability of young people with migraine to depression and suicidal ideation is particularly worrying. For both genders, migraineurs under the age of 30 had at least six times the odds of current depression and four times the odds of lifetime suicidal ideation when compared to those aged 65 and above. First onset of migraine is typically experienced in late adolescence and early adulthood [23]. The diagnosis of migraine at a young age may interfere with normal developmental processes, such as obtaining an education, building a career, and starting a family, which may contribute to the experience of depression and suicidal ideation [26]. Older migraineurs, by contrast, have had a longer time to adjust to their condition, for example, by learning effective coping mechanisms or achieving adequate treatment, which may reduce the perceived burden of their illness. 

The finding that single people with migraine have between 50 and 70% higher odds of depression and suicidal ideation also deserves some attention. The inverse relationship between social support and depression is well established [27]. Social support may be particularly important in the context of severe or uncontrollable pain [28, 29]. There is some evidence that those who have a hard time controlling their pain receive less social support than they desire, which contributes to depression [28]. 

The strong relationship found between the extent of disability and both depression and suicidal ideation among those with migraine is also notable. For example, migraineurs who often have difficulties with activities were shown to have approximately three times the odds of both depression and suicidal ideation. Note that many of the dimensions of disability assessed in the current study (e.g., difficulty seeing, hearing, communicating, and walking) can be considered part of the migraine spectrum (e.g., photophobia, phonophobia, visual aura, sensible aura, motor aura, and dysarthric aura). A high level of disability may reflect unmet treatment needs, which also play a role in depressed mood [30]. Thus, achieving optimal management of migraine should be a primary focus of care. Unlike age, both lack of social support and unmet treatment needs are modifiable factors and, thus, can be affected by intervention. Recently, a web-based program designed to increase self-efficacy in migraine management and reduce distress was shown to increase headache self-efficacy, increase the use of relaxation techniques and social support, and decrease pain catastrophizing, depression, and stress [31]. Further research should test whether this type of intervention, which is highly accessible, including to those outside of the healthcare system, should become a routine part of the management of migraine headaches. 

It is already recommended that those with migraine are screened for depression [6]. The elevated rates of lifetime suicidal ideation found among both men and women with migraine suggest that this should include an assessment of suicide risk, although further research is needed to establish whether routine screening of this sort is effective. Educating general practitioners to better identify and treat depression may also help reduce suicidal ideation while enhancing well-being [25]. As many of those with migraine may not have had a recent consultation with a medical doctor about their headaches [23], there is a need to increase opportunities for identification and treatment of depression outside of primary care. Informing a wider range of health professionals and migraine sufferers themselves about the patterns of depression and suicidal ideation surrounding age, marital status, and activity limitations may help to increase awareness of the comorbidities of migraine and empower migraineurs to come forward with their mental health concerns. 

This study has at least four limitations that should be considered when interpreting the results. First, the presence of migraine was based on the respondent's report of a health professional's diagnosis. While previous research shows that the accuracy of a self-reported history of physician-diagnosed chronic health conditions can be quite good [32, 33], this does not guarantee that all those with migraine actually were captured. Research shows that almost one third of adults with migraine have never seen a doctor for their migraine [23], and, thus, a proportion of respondents may have met the clinical criteria for migraine, but have never received a diagnosis. The high potential for underreporting of migraine suggests, however, that if misclassifications were to occur, they would most likely render the results more conservative. 

Second, this study could not differentiate between different types of migraine. Research suggests that migraine with aura has a stronger relationship with depression and maybe suicidal behaviours than migraine without aura [8, 19]. Chronic migraines are also found to be more highly associated with depression than episodic migraines [30]. It is possible, therefore, that factors associated with depression and suicidal ideation also vary according to the type of migraine. The investigation of other migraine-specific determinants in relation to depression and suicidal ideation would also be helpful, such as migraine attack frequency, cutaneous allodynia, and sleep disorders. 

Third, this study did not control for other pain conditions or anxiety disorders, both of which are associated with depression and migraine [11, 12, 19]. While it is not expected that pain or anxiety would explain the entire relationship between migraine and depression or suicidal ideation, they could influence the extent to which other factors, such as age, income, marital status, and disability, are associated with depression/suicidal ideation among those with migraine. Finally, this study was based on cross-sectional data, which means that it could not investigate the bidirectional nature of the relationship between each of depression and suicidal ideation and migraine. It also could not determine whether the factors associated with depression/suicidal ideation among those with migraine are predictors or outcomes. For example, low income could trigger the onset of depression among those with migraine, or it could be an outcome of having comorbid migraine and depression, perhaps relating to the difficulties of maintaining paid work. Longitudinal research is needed to better understand how these complex relationships unfold over time. 

## 5. Conclusion

This study confirms that migraine is associated with higher odds of current depression and lifetime suicidal ideation among Canadian men and women living in the community. Among those with migraine, it identifies the gender-specific factors that are independently associated with depression/suicidal ideation. It is already recommended that all those with migraine are screened for depression. The results of this study can be used to help identify the migraineurs who may require the most immediate attention, including those who are younger, unmarried, and experiencing limitations in their activities. 

## Figures and Tables

**Table 1:** Gender-specific odds of depression for those with migraine versus those without.

Variables	Women (*n* = 36,176)						Men (*n* = 30,544)					
	Model 1		Model 2		Model 3		Model 1		Model 2		Model 3	
	OR	95% CI	OR	95% CI	OR	95% CI	OR	95% CI	OR	95% CI	OR	95% CI
Has migraine headaches												
No	1.00	Referent	1.00	Referent	1.00	Referent	1.00	Referent	1.00	Referent	1.00	Referent
Yes	2.17	(1.97, 2.40)	2.17	(1.96, 2.40)	1.89	(1.71, 2.10)	2.49	(2.10, 2.95)	2.46	(2.07, 2.91)	2.02	(1.70, 2.41)
*Demographics *												
Age												
<30	4.07	(3.31, 5.01)	4.29	(3.46, 5.31)	6.86	(5.50, 8.57)	2.82	(2.10, 3.79)	2.25	(1.65, 3.07)	3.83	(2.79, 5.25)
30–49	3.46	(2.82, 4.26)	4.63	(3.73, 5.74)	6.67	(5.35, 8.31)	3.80	(2.85, 5.06)	4.32	(3.22, 5.80)	6.27	(4.65, 8.45)
50–64	2.71	(2.18, 3.37)	3.50	(2.80, 4.37)	4.34	(3.47, 5.44)	2.39	(1.76, 3.24)	2.75	(2.02, 3.74)	3.28	(2.41, 4.48)
65+	1.00	Referent	1.00	Referent	1.00	Referent	1.00	Referent	1.00	Referent	1.00	Referent
Race												
White	1.10	(0.96, 1.25)	1.14	(1.00, 1.29)	1.12	(0.99, 1.28)	1.25	(1.04, 1.49)	1.32	(1.11, 1.58)	1.24	(1.03, 1.49)
Visible minority	1.00	Referent	1.00	Referent	1.00	Referent	1.00	Referent	1.00	Referent	1.00	Referent
*Adult SES and Marital Status *												
Household income												
Missing			0.84	(0.73, 0.97)	0.82	(0.72, 0.95)			0.94	(0.78, 1.15)	0.92	(0.75, 1.12)
0–29,999			1.56	(1.39, 1.74)	1.38	(1.23, 1.54)			1.85	(1.60, 2.15)	1.61	(1.38, 1.87)
30,000 or more			1.00	Referent	1.00	Referent			1.00	Referent	1.00	Referent
Education												
≤High school degree			0.96	(0.87, 1.05)	0.93	(0.84, 1.02)			0.88	(0.78, 1.00)	0.82	(0.72, 0.93)
Postsecondary degree			1.00	Referent	1.00	Referent			1.00	Referent	1.00	Referent
Marital status												
Not married			1.73	(1.57, 1.91)	1.68	(1.52, 1.86)			2.10	(1.84, 2.40)	2.08	(1.82, 2.38)
Married			1.00	Referent	1.00	Referent			1.00	Referent	1.00	Referent
*Disability *												
Has difficulty with activities												
Sometimes					2.11	(1.87, 2.38)					2.80	(2.41, 3.26)
Often					3.14	(2.75, 3.58)					3.58	(3.04, 4.23)
Never					1.00	Referent			1.00	Referent	1.00	Referent
ADL												
No					1.00	Referent			1.00	Referent	1.00	Referent
Yes					2.02	(1.55, 2.64)					2.29	(1.65, 3.20)

ADL = Activities of daily living.

**Table 2:** Gender-specific crude and adjusted odds of depression among those with migraines.

	Women (*n* = 4812^†^)				Men (*n* = 1765^†^)			
	Crude		Adjusted		Crude		Adjusted	
	OR	95% CI	OR	95% CI	OR	95% CI	OR	95% CI
Age								
<30	4.49	(2.28, 8.84)	7.73	(3.83, 15.62)	3.25	(0.95, 11.06)	6.00	(1.67, 21.65)
30–49	4.00	(2.05, 7.82)	7.48	(3.73, 15.00)	3.41	(1.01, 11.55)	6.42	(1.85, 22.33)
50–64	3.63	(1.83, 7.22)	5.51	(2.72, 11.17)	4.40	(1.28, 15.10)	6.21	(1.76, 21.86)
65+	1.00	Referent	1.00	Referent	1.00	Referent	1.00	Referent
Race								
White	0.92	(0.71, 1.19)	1.04	(0.79, 1.36)	0.73	(0.47, 1.13)	0.87	(0.53, 1.40)
Visible minority	1.00	Referent	1.00	Referent	1.00	Referent	1.00	Referent
Household income								
Missing	0.89	(0.67, 1.17)	0.83	(0.62, 1.12)	1.72	(1.07, 2.76)	1.64	(0.99, 2.74)
0–29,999	1.82	(1.49, 2.23)	1.43	(1.14, 1.80)	3.76	(2.62, 5.39)	2.69	(1.80, 4.02)
30,000 or more	1.00	Referent	1.00	Referent	1.00	Referent	1.00	Referent
Education								
≤High school degree	1.12	(0.94, 1.33)	1.04	(0.86, 1.26)	1.13	(0.82, 1.55)	0.87	(0.61, 1.25)
Postsecondary degree	1.00	Referent	1.00	Referent	1.00	Referent	1.00	Referent
Marital status								
Not married	1.87	(1.57, 2.22)	1.71	(1.40, 2.08)	1.68	(1.22, 2.31)	1.53	(1.03, 2.27)
Married	1.00	Referent	1.00	Referent	1.00	Referent	1.00	Referent
Difficulty with activities								
Sometimes	1.96	(1.56, 2.45)	1.96	(1.55, 2.48)	4.61	(3.15, 6.74)	3.96	(2.64, 5.93)
Often	3.07	(2.46, 3.82)	3.03	(2.38, 3.86)	3.53	(2.36, 5.28)	3.20	(2.05, 4.99)
Never	1.00	Referent	1.00	Referent	1.00	Referent	1.00	Referent
ADL								
No	1.00	Referent	1.00	Referent	1.00	Referent	1.00	Referent
Yes	3.47	(2.30, 5.22)	2.20	(1.40, 3.45)	6.87	(3.54, 13.35)	3.01	(1.43, 6.33)

^†^Sample size of the adjusted analysis.

ADL = Activities of daily living.

**Table 3:** Gender-specific odds ratio of lifetime suicidal ideation among those with migraine versus those without.

Variables	Women (*n* = 44,600)						Men (*n* = 36,868)					
	Model 1		Model 2		Model 3		Model 1		Model 2		Model 3	
	OR	95% CI	OR	95% CI	OR	95% CI	OR	95% CI	OR	95% CI	OR	95% CI
*Exposure Variable *												
Has migraine headaches												
No	1.00	Referent	1.00	Referent	1.00	Referent	1.00	Referent	1.00	Referent	1.00	Referent
Yes	1.95	(1.81, 2.10)	1.93	(1.79, 2.08)	1.72	(1.59, 1.86)	2.01	(1.79, 2.25)	2.01	(1.79, 2.25)	1.70	(1.55, 1.96)
*Demographics *												
Age												
<30	2.72	(2.38, 3.11)	2.99	(2.61, 3.44)	4.38	(3.79, 5.05)	2.42	(2.07, 2.84)	2.06	(1.74, 2.44)	3.03	(2.55, 3.60)
30–49	2.51	(2.21, 2.86)	3.57	(3.12, 4.09)	4.80	(4.17, 5.52)	2.49	(2.14, 2.91)	2.86	(2.44, 3.35)	3.78	(3.22, 4.45)
50–64	2.43	(2.12, 2.78)	3.24	(2.82, 3.72)	3.85	(3.34, 4.44)	2.39	(2.04, 2.81)	2.78	(2.36, 3.27)	3.25	(2.75, 3.83)
65+	1.00	Referent	1.00	Referent	1.00	Referent	1.00	Referent	1.00	Referent	1.00	Referent
Race												
White	1.51	(1.37, 1.66)	1.60	(1.45, 1.77)	1.58	(1.43, 1.74)	1.82	(1.62, 2.04)	1.89	(1.69, 2.13)	1.81	(1.61, 2.04)
Visible minority	1.00	Referent	1.00	Referent	1.00	Referent	1.00	Referent	1.00	Referent	1.00	Referent
*Adult SES and Marital Status *												
Household income												
Missing			0.97	(0.88, 1.08)	0.95	(0.85, 1.05)			0.85	(0.74, 0.97)	0.83	(0.73, 0.95)
0–29,999			1.65	(1.52, 1.80)	1.49	(1.36, 1.62)			1.79	(1.62, 1.97)	1.61	(1.46, 1.79)
30,000 or more			1.00	Referent	1.00	Referent			1.00	Referent	1.00	Referent
Education												
≤High school degree			1.10	(1.03, 1.18)	1.08	(1.01, 1.16)			0.97	(0.90, 1.04)	0.91	(0.85, 0.99)
Postsecondary degree			1.00	Referent	1.00	Referent			1.00	Referent	1.00	Referent
Marital status												
Not married			1.59	(1.48, 1.71)	1.55	(1.44, 1.67)			1.76	(1.61, 1.91)	1.75	(1.60, 1.90)
Married			1.00	Referent	1.00	Referent			1.00	Referent	1.00	Referent
*Disability *												
Has difficulty with activities												
Sometimes					2.01	(1.84, 2.20)					2.29	(2.08, 2.52)
Often					2.73	(2.47, 3.02)					2.83	(2.54, 3.15)
Never					1.00	Referent					1.00	Referent
ADL												
No					1.00	Referent					1.00	Referent
Yes					1.30	(1.06, 1.59)					1.79	(1.41, 2.28)

ADL = Activities of daily living.

**Table 4:** Gender-specific crude and adjusted odds ratio of lifetime suicidal ideation among migraineurs.

	Women (*n* = 6,340^†^)				Men (*n* = 2,255^†^)			
	Crude		Adjusted		Crude		Adjusted	
	OR	95% CI	OR	95% CI	OR	95% CI	OR	95% CI
Age								
<30	2.61	(1.38, 4.92)	4.25	(2.17, 8.31)	3.40	(1.02, 11.29)	4.43	(1.25, 15.66)
30–49	2.51	(1.35, 4.67)	4.58	(2.38, 8.83)	3.96	(1.21, 12.94)	5.71	(1.70, 19.18)
50–64	2.24	(1.18, 4.27)	3.40	(1.74, 6.65)	3.35	(0.99, 11.35)	3.67	(1.06, 12.73)
65+	1.00	Referent	1.00	Referent	1.00	Referent	1.00	Referent
Race								
White	1.17	(0.87, 1.57)	1.31	(0.97, 1.78)	2.72	(1.43, 5.19)	2.96	(1.52, 5.78)
Visible minority	1.00	Referent	1.00	Referent	1.00	Referent	1.00	Referent
Household income								
Missing	1.30	(0.97, 1.74)	1.22	(0.89, 1.68)	0.68	(0.36, 1.27)	0.62	(0.31, 1.24)
0–29,999	1.79	(1.40, 2.30)	1.44	(1.09, 1.91)	1.20	(0.76, 1.88)	1.07	(0.64, 1.79)
30,000 or more	1.00	Referent	1.00	Referent	1.00	Referent	1.00	Referent
Education								
Not postsecondary	1.10	(0.90, 1.35)	1.03	(0.82, 1.28)	1.14	(0.82, 1.58)	1.05	(0.73, 1.52)
Postsecondary grad	1.00	Referent	1.00	Referent	1.00	Referent	1.00	Referent
Marital status								
Not married	1.74	(1.42, 2.13)	1.60	(1.26, 2.02)	1.43	(1.03, 2.00)	1.60	(1.05, 2.44)
Married	1.00	Referent	1.00	Referent	1.00	Referent	1.00	Referent
Difficulty with activities								
Sometimes	1.59	(1.22, 2.07)	1.64	(1.25, 2.16)	3.47	(2.32, 5.20)	3.60	(2.36, 5.48)
Often	2.91	(2.24, 3.78)	2.97	(2.22, 3.97)	2.68	(1.75, 4.11)	2.87	(1.79, 4.61)
Never	1.00	Referent	1.00	Referent	1.00	Referent	1.00	Referent
ADL								
No	1.00	Referent	1.00	Referent	1.00	Referent	1.00	Referent
Yes	1.70	(1.01, 2.88)	1.08	(0.62, 1.90)	3.05	(1.28, 7.26)	2.04	(0.79, 5.26)

^†^Sample size of the adjusted analysis.

ADL = Activities of daily living.

## References

[1] American Migraine Foundation 36 million Migraine Campaign. https://www.americanmigrainefoundation.org/assets/1/7/36millionmigrainecampaign_v4.pdf.

[2] Freitag FG (2007). The cycle of migraine: patients’ quality of life during and between migraine attacks. *Clinical Therapeutics*.

[3] Lipton RB, Stewart WF, Diamond S, Diamond ML, Reed M (2001). Prevalence and burden of migraine in the United States: data from the American Migraine Study II. *Headache*.

[4] Buse DC, Lipton RB (2013). Global perspectives on the burden of episodic and chronic migraine. *Cephalalgia*.

[5] Gilmour H, Wilkins K (2001). Migraine. *Health Report*.

[6] Hamelsky SW, Lipton RB (2006). Psychiatric comorbidity of migraine. *Headache*.

[7] Stewart WF, Ricci JA, Chee E, Morganstein D, Lipton R (2003). Lost productive time and cost due to common pain conditions in the US workforce. *Journal of the American Medical Association*.

[8] Breslau N, Schultz LR, Stewart WF, Lipton RB, Lucia VC, Welch KMA (2000). Headache and major depression: is the association specific to migraine?. *Neurology*.

[9] Jette N, Patten S, Williams J, Becker W, Wiebe S (2008). Comorbidity of migraine and psychiatric disorders—a national population-based study. *Headache*.

[10] Lipton RB, Hamelsky SW, Kolodner KB, Steiner TJ, Stewart WF (2000). Migraine, quality of life, and depression: a population-based case-control study. *Neurology*.

[11] Zwart J-A, Dyb G, Hagen K (2003). Depression and anxiety disorders associated with headache frequency. The Nord-Trøndelag Health Study. *European Journal of Neurology*.

[12] McWilliams LA, Goodwin RD, Cox BJ (2004). Depression and anxiety associated with three pain conditions: results from a nationally representative sample. *Pain*.

[13] Ratcliffe GE, Enns MW, Jacobi F, Belik S-L, Sareen J (2009). The relationship between migraine and mental disorders in a population-based sample. *General Hospital Psychiatry*.

[14] Victor TW, Hu X, Campbell J, White RE, Buse DC, Lipton RB (2010). Association between migraine, anxiety and depression. *Cephalalgia*.

[15] Breslau N, Lipton RB, Stewart WF, Schultz LR, Welch KMA (2003). Comorbidity of migraine and depression: investigating potential etiology and prognosis. *Neurology*.

[16] Schur EA, Noonan C, Buchwald D, Goldberg J, Afari N (2009). A twin study of depression and migraine: evidence for a shared genetic vulnerability. *Headache*.

[17] Stam AH, De Vries B, Janssens ACJW (2010). Shared genetic factors in migraine and depression: evidence from a genetic isolate. *Neurology*.

[18] Breslau N, Davis GC, Andreski P (1991). Migraine, psychiatric disorders, and suicide attempts: an epidemiologic study of young adults. *Psychiatry Research*.

[19] Ratcliffe GE, Enns MW, Belik S-L, Sareen J (2008). Chronic pain conditions and suicidal ideation and suicide attempts: an epidemiologic perspective. *Clinical Journal of Pain*.

[20] Breslau N, Schultz L, Lipton R, Peterson E, Welch KMA (2012). Migraine Headaches and Suicide Attempt. *Headache*.

[21] Statistics Canada Canadian Community Health Survey (CCHS). http://www23.statcan.gc.ca/imdb/p2SV.pl?Function=getSurvey&SurvId=3226&SurvVer=0&InstaId=15282&InstaVer=3&SDDS=3226&lang=en&db=imdb&adm=8&dis=2.

[22] Kessler RC, Andrews G, Mroczek D, Ustun B, Wittchen HU (1998). The World Health Organization Composite International Diagnostic Interview Short-Form (CIDI-SF). *International Journal of Methods in Psychiatric Research*.

[23] Lipton RB, Scher AI, Kolodner K, Liberman J, Steiner TJ, Stewart WF (2002). Migraine in the United States: epidemiology and patterns of health care use. *Neurology*.

[24] Taylor RR, Jason LA, Jahn SC (2003). Chronic fatigue and sociodemographic characteristics as predictors of psychiatric disorders in a community-based sample. *Psychosomatic Medicine*.

[25] U.S. Preventive Services Task Force Screening for suicide risk: a systematic evidence review for the U.S. Preventive Services Task Force. http://www.ahrq.gov/downloads/pub/prevent/pdfser/suicidser.pdf.

[26] Helvig AW, Minick P (2013). Adolescents and headaches: maintaining control. *Pediatric Nursing*.

[27] Kawachi I, Berkman LF (2001). Social ties and mental health. *Journal of Urban Health*.

[28] Cho S, Zunin ID, Chao PJ, Heiby EM, McKoy J (2012). Effects of pain controllability and discrepancy in social support on depressed mood among patients with chronic pain. *International Journal of Behavioral Medicine*.

[29] Fuller-Thomson E, Sulman J (2006). Depression and inflammatory bowel disease: findings from two nationally representative Canadian surveys. *Inflammatory Bowel Diseases*.

[30] Blumenfeld AM, Varon SF, Wilcox TK (2011). Disability, HRQoL and resource use among chronic and episodic migraineurs: results from the International Burden of Migraine Study (IBMS). *Cephalalgia*.

[31] Bromberg J, Wood ME, Black RA, Surette DA, Zacharoff KL, Chiauzzi EJ (2012). A randomized trial of a web-based intervention to improve migraine self-management and coping. *Headache*.

[32] Okura Y, Urban LH, Mahoney DW, Jacobsen SJ, Rodeheffer RJ (2004). Agreement between self-report questionnaires and medical record data was substantial for diabetes, hypertension, myocardial infarction and stroke but not for heart failure. *Journal of Clinical Epidemiology*.

[33] Oksanen T, Kivimäki M, Pentti J, Virtanen M, Klaukka T, Vahtera J (2010). Self-report as an indicator of incident disease. *Annals of Epidemiology*.

